# Recycling and Reusing of Waste Aircraft Composites in Thermoplastic and Thermoset Matrices

**DOI:** 10.3390/ma19030534

**Published:** 2026-01-29

**Authors:** Paulina Latko-Durałek, Kamila Sałasińska, Bartłomiej Bereska, Agnieszka Bereska, Anna Czajka-Warowna, Paweł Durałek, Maria Kosarli, Alexia Koutrakou, Michał Sałaciński, Gaylord Booto, Sotirios Grammatikos

**Affiliations:** 1 TMBK Partners Sp. z o.o., ul. Bitwy Warszawskiej 1920 r. 7A, 02-366 Warsaw, Poland; pawel.duralek@tmbk.pl; 2 Faculty of Materials Science and Engineering, Warsaw University of Technology, ul. Wołoska 141, 02-507 Warsaw, Poland; kamila.salasinska@pw.edu.pl (K.S.); anna.czajka@pw.edu.pl (A.C.-W.); 3 NOMA Resins Sp. z o.o., ul. Bojkowska 35A, 44-100 Gliwice, Poland; b.bereska@noma.com.pl (B.B.); a.bereska@noma.com.pl (A.B.); 4 Global Consulting Sustainability AS, Buskerudveien 33, 3024 Drammen, Norway; m.kosarli@globalconsultingsustainability.com (M.K.); alexkoutrakou@gmail.com (A.K.); contact@globalconsultingsustainability.com (G.B.); 5 Air Force Institute of Technology, ul. Księcia Bolesława 6, 01-494 Warsaw, Poland; michal.salacinski@itwl.pl; 6 ASEMlab—Laboratory of Advanced & Sustainable Engineering Materials, Norwegian University of Science and Technology—NTNU, Teknologivegen 22, 2815 Gjøvik, Norway; sotirios.grammatikos@ntnu.no

**Keywords:** fiber-reinforced polymers, recycling, epoxy, polyamide, thermal properties, mechanical properties, life cycle assessment

## Abstract

Unlike typical fiber-reinforced polymers, aerospace composites consist of 90% carbon and 10% glass fabrics impregnated with thermosetting resin. Due to the strong bonding between fibers and the thermoset nature of the matrix, recycling these materials is particularly challenging. This study evaluates mechanical recycling of aircraft composite waste via industrial grinding and chemical recycling through a solvolysis process. Recovered fibrous fractions were integrated into an epoxy matrix at 50 wt% loading using hot-pressing and into polyamide 12 at 15 wt% via a twin-screw extrusion process. The mechanical results showed that chemically recycled fibers in epoxy reached a flexural modulus of 9.9 GPa and strength of 112 MPa, significantly outperforming mechanically recycled fillers (6.1 GPa and 98.0 MPa) compared to virgin carbon fibers (11.3 GPa and 132 MPa). In PA12, the addition of chemically recycled fibers yielded a 2.14 GPa modulus and a 67.7 MPa strength. Furthermore, life cycle assessment confirmed that both recycling routes drastically reduce global warming potential and aquatic ecotoxicity compared to landfilling. These findings indicate that while mechanical recycling is simpler, chemical solvolysis provides a superior pathway for the high-value circular reuse of complex aerospace waste in new thermoplastic and thermoset applications.

## 1. Introduction

Carbon- and glass-fiber reinforced polymers (CFRP and GFRP) are extensively employed in the aviation and automotive industries due to their superior strength, stiffness, fatigue resistance, and lightweight properties. They account for about 50% of the Boeing 787’s structure, including the fuselage, wings, and doors [1]. The application of lightweight composites results in average weight savings of around 20%, reducing maintenance costs and lowering fuel consumption [1].

The International Civil Aviation Organization estimated that 600–1000 aircraft are retired worldwide yearly and that this number could reach 15,000 by 2040. Consequently, the global aircraft recycling market is expected to grow from $4.74 billion in 2023 to $9.7 billion in 2028 [2]. Currently, aircraft recovery rates commonly reach 80–85%, mainly concerning engines and other metal parts. Almost 50% of the materials, mainly metals, can be recycled, but around 40% of the parts are reused in other applications or as spare parts. The key challenge for achieving 100% recycling is polymer-based composites like CFRP and GFRP, which are hard to recycle due to their short lifespan and complex, costly processes, making storage a simpler option [3,4]. Recycling composite materials poses significant challenges due to their composition of chemically diverse components. For example, typical structural composites used in aircraft consist of thermosetting resin, polymeric foams, organic fibers, and inorganic paint, all of which need to be separated before recycling because of their diverse chemical natures. Among these components, the most problematic are thermosetting resins (e.g., epoxy or polyester) because they cannot be reformed due to the presence of a crosslinked network. Additionally, all current recovery methods result in reduced strength and mechanical properties, diminishing the value of the recovered materials [5,6].

Three main recycling methods are used, depending on the properties and applications of the polymer composites: mechanical, chemical, and thermal recycling. Mechanical recycling consists of crushing the waste to reduce its size and using it as raw material for another product. It is the simplest method that can be easily implemented, with low CO_2_ emissions and energy consumption. However, the obtained fraction contains not only the fiber fragments but also the resin scraps, which stick to the fibers, reducing their mechanical properties. Therefore, firstly, the composite parts are shredded to obtain a 50–100 mm fraction, and then they are milled to a smaller size recyclate with 10–50 mm, after which the resin scraps are sieved to separate them from the fibers [7,8]. Most of the research in mechanical recycling deals with process optimization to obtain fractions homogeneous in size, without traces of cured resins, and to reduce the processing cost. There are companies that specialize in the mechanical recycling of polymer composites, but mainly there are simple composites made by sheet- and bulk molding compound (SMC or BMC), which consist of only thermoset resin and fibers [9,10]. Thermal recycling uses pyrolysis to degrade composite waste in an oxygen-free environment, separating the matrix into organic liquids, gases, and small compounds. It efficiently reclaims short fibers with minimal resin but emits high CO_2_ levels, consumes significant energy, and may damage fiber surfaces due to overheating [11,12]. Chemical recycling degrades polymer waste into monomers or simple chemicals, with recovered fibers used as fillers and hydrocarbons as fuel or raw materials. However, this method requires solvents, generates many by-products, and is energy intensive [6]. Research focuses on solvolysis, glycolysis, and hydrolysis, which vary by reactive medium, with outcomes influenced by temperature (usually under 400 °C) and pressure. Zhao et al. [13] proposed a two-step recycling process for amine-cured CFRP that consists of swelling in acetic acid at 90 °C for 40 min (pretreatment) and solvolysis in monoethanolamine containing 10 wt% potassium hydroxide at 160 °C for 90 min. The degradation ratio of the composite samples reached up to 99%. Recycled carbon fibers recovered from this treatment maintained above 93% of their original tensile strength and elastic modulus. In the other approach, CFRP was immersed in nitric acid at 80 °C for 8 h, and then in sodium hydrogen carbonate at 80 °C for 15 min. This environmentally friendly process enables the recovery of carbon fibers with improved physical properties compared to virgin carbon fibers and possibly the recovery of decomposed epoxy resin from CFRP [14]. Kim et al. [15] investigated the use of potassium permanganate solution for the oxidative depolymerization of CFRP to recover clean carbon fibers exhibiting good mechanical properties (91% of the value of the virgin fibers) and reached degradation up to 94%. In the other study [16], acetic acid solution under subcritical conditions was used to recover clean, high-quality recycled carbon fibers. The process, conducted in a high-pressure batch reactor at 300 °C for 30 min, involved composite swelling, matrix depolymerization, and decomposition. Heating caused autogenic pressure to rise to 10 MPa, achieving up to 100% fiber recovery efficiency.

Recycling procedures for specific materials should also account for reusing recycled products in new applications. For single-use plastics, mechanical recycling produces pellets that can be processed into new products with or without virgin polymer. In contrast, CFRP and GFRP consist of diverse components and require chemical or thermal recycling to separate fibers from cured resin. Recycled glass and carbon fibers are already available in forms like short fibers, nonwoven fabrics, and mats, which can reinforce thermoset and thermoplastic polymers, concrete, and gypsum or serve as toughening interlayers for fiber-reinforced polymers [8,17,18,19,20]. It has been shown that recycled materials can enhance the mechanical properties of unmodified materials, aligning with sustainable development principles [21]. Another approach involves using CFRP/GFRP waste from mechanical recycling without separating fibers from the resin. The recyclate’s concentration and size are crucial for improving mechanical properties, as demonstrated with epoxy and polyester resins [22]. Mechanically recycled CFRP has also been tested as reinforcement for epoxy foam, significantly increasing compressive modulus and strength [23], and as an effective filler in acrylonitrile-butadiene-styrene, reducing electrical resistance of polymer [24].

The development of the recycling method of composites is related to the fact that most of the composite waste is landfilled or incinerated. However, there is a need to evaluate the strengths and weaknesses of recycling methods before their industrial implementation by conducting a life cycle assessment (LCA) as the basic tool for specific materials and regions [17]. LCA is a systematic and comprehensive method for the evaluation of the environmental impacts of a product, process, or service throughout its entire life cycle. This includes raw material extraction, material processing, manufacturing, distribution, use, repair and maintenance, and disposal or recycling. By examining each stage, LCA helps identify opportunities for improving environmental performance and reducing the overall ecological footprint [18]. The performed LCA studies of polymer composites have consistently shown that, from an environmental perspective, recycling is the most beneficial end-of-life scenario, and the produced products can be reused in new application areas [19].

This paper investigates the application of mechanical and chemical recycling routes to complex hybrid composite waste generated by an aerospace materials manufacturer. The waste consists of glass- and carbon-fiber-reinforced composites in which the two fiber types are intimately bonded and cannot be readily separated. However, most of the paper deals with the mechanical and chemical recycling of pure CFRP or GFRP [7,10,21]. To the authors’ knowledge, the recycling of such hybrid CFRP/GFRP waste has not been previously addressed in the literature, which underlines the novelty of the present study. Furthermore, the paper proposes potential valorization pathways for the recycled materials in both thermoset and thermoplastic polymer matrices and evaluates the properties of composites based on epoxy resin and polyamide 12. The assessment of the mechanical and chemical recycling strategies is complemented by a life cycle assessment to quantify their environmental performance.

## 2. Materials and Methods

### 2.1. Recycling Process of Composite Waste

**Figure 1:**
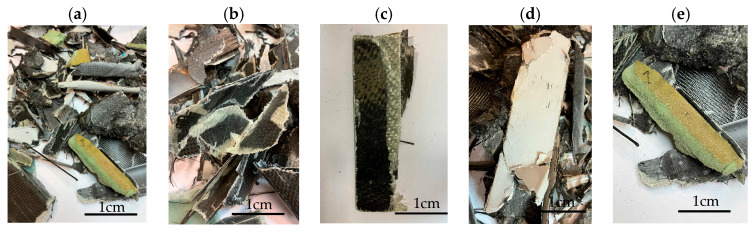
Starting hybrid composite waste (CGFRP) used in recycling trials: general mixture of the components (**a**,**b**); carbon and glass fabrics bonded together (**c**); paint (**d**); and polymer foam (**e**).

In this work we studied post-production fiber-reinforced composites as waste material provided by WZL, Dęblin, Poland, which specializes in manufacturing structural composites for aircraft. Composite waste was shredded to irregular shapes with various sizes (from 10 to 100 mm) using industrial grinding machines. According to the producer, the composites contain mainly carbon fiber (90 wt%) but also glass fiber reinforcement (10 wt%), which are used as the upper finish layer of the composite. Moreover, the waste provided included cured epoxy resin, paint, and foam, as can be seen in the photos shown in Figure 1. The remaining foam was separated manually and not used further in the recycling process. It was not possible to disconnect/dismantle the glass fabric from the carbon fabric, cured resin, or paint, so they were processed together by mechanical and chemical recycling.

Mechanical recycling performed by TMBK Partners Sp. z o.o. (Warsaw, Poland) was conducted using a grinder, which allowed grinding the shredded waste into shorter fragments and traces of cured resin and paint. Fiber fragments (fibrous fragments, which are lumps of fiber reinforcement mixed with cured resin) obtained by mechanical recycling were designed as *m*CF/GF, and they were not separated from resin. Chemical recycling was performed by NOMA Resins Sp. z o.o. (Gliwice, Poland). The composite waste (shown in Figure 1) was immersed in a three-necked flask containing an aqueous nitric acid solution (4 M). The flask was placed in a constant temperature bath at 80 °C, and the epoxy resin was decomposed by the HNO_3_ over 4 h, producing a yellow solution. The solid residue, containing carbon and glass fibers, was collected from the flask and rinsed using deionized water. Next, the fibers were dried (at 80 °C for 48 h) to remove any remaining moisture. Figure S1 in the Supplementary Material shows the laboratory set-up for the solvolysis process, recycled fibers after chemical recycling, and the obtained extract. All reagents used for chemical recycling were procured from Chempur (Piekary Śląskie, Poland). Fibers obtained by chemical recycling were designated as *ch*CF/GF.

The fibrous fragments obtained by mechanical recycling (*m*CF/GF) and the glass/carbon fiber mixture obtained by chemical recycling (*ch*CF/GF) are listed in Table 1 together with their macro- and microstructures. SEM images of the fillers after mechanical and chemical recycling reveal their heterogeneous morphology. Crushed resin particles and micro-composite fragments are visible in both mechanically and chemically recycled fibers. The white fibers observed in the images correspond to glass fibers. These recyclates were used as fillers for two polymer matrices: a thermoset and a thermoplastic. For epoxy-based composites processed by hot-pressing, the fillers were used as received. For polyamide-based composites processed by extrusion, the composite waste was milled by a ultracentrifugal (Restsch ZM200, Restsch GmbH, Haan, Germany) mill at 18,000 rpm and using a sieve with a mesh size of 0.25 mm. For comparison, virgin carbon fibers (*v*CF) supplied by SGL Carbon (Wiesbaden, Germany) with a diameter of 13 µm (for thermoset composites) and after milling to 0.25 mm (for thermoplastic composites) were used as reference fillers.

### 2.2. Composites Manufacturing

**Figure 2:**
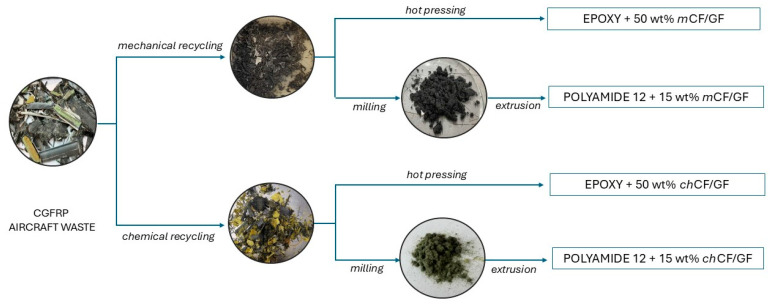
The schematic approach applied for composite fabrication.

Thermosetting and thermoplastic composites were manufactured according to the graphical procedure presented in Figure 2. The list of prepared composites is presented in Table 2.

Thermoset composites were fabricated using the Noma Comp MV/MRA epoxy resin system (NOMA Resins Sp. z o.o., Gliwice, Poland). Noma Comp MV is an epoxy resin characterized by a viscosity in the range of 2.5–3.0 Pa·s and an epoxy equivalent weight (EEW) between 184 and 194 g·eq^−1^. Each type of fiber (*m*CF/GF and *ch*CF/GF), after recycling, was pre-mixed with the epoxy formulation at a concentration of 50 wt% at room temperature until complete resin uptake by the fibers. Such a high content of waste was added because it is a standard loading in epoxy-based composites production to achieve a high-performance material. The resin–fiber mixtures were poured into a steel mold with dimensions of 100 × 100 × 4 mm and cured in a hot press (PHM-250E, Ponar Sp. z o.o., Żywiec, Poland) under a pressure of 5.0 MPa according to the following procedure: the mold was inserted into the press with heated plates set to 80 °C and held at constant temperature for 120 min while applying a pressure of 4.0 MPa. The sample was then maintained under these isothermal conditions for an additional 240 min. Finally, before opening the hot-press plates, the mold was cooled to room temperature. The obtained composite panels were cut using a CNC machine into specimens with dimensions of 80 × 10 × 4 mm (Figure S2 in the Supplementary Material) for thermal and mechanical characterization.

Thermoplastic composites were prepared with the same type of fibers *(v*CF, *m*CF/GF, and *ch*CF/GF) but with a percentage of 15 wt%. Polyamide 12, which is characterized by a density of 1.01 g/cm^3^, water absorption of 1.5%, a melting point of 179 °C, and a processing temperature between 190 and 240 °C, was used as the matrix (Vestamid^®^Z7321, Evonik, Essen, Germany). The pellets of PA12 were initially dried in a vacuum oven at 90 °C for at least 24 h before further use. Contrary to the thermosetting composites, in this case, all the types of fibers were additionally ground, as presented in Table 1. Polymer and filler with a concentration of 15 wt% were mixed together using the twin-screw extruder HAAKE MiniLab (ThermoFisher Scientific, Waltham, MA, USA) at 195 °C, with the screws rotating at 25 rpm and a mixing time (residence time) of 5 min. The fabricated pellets were then processed into the rectangular specimens (Figure S2 in the Supplementary Material) needed for the 3-point bending test (80 mm × 10 mm × 4 mm) using a HAAKE Mini Jet Piston Injection Molding System (ThermoFisher Scientific, MA, USA). The parameters of the injection molding were as follows: 220–225 °C—the temperature of the barrel; 50–60 °C—the mold temperature; 700–800 bars and 8–10 s—the injection pressure and time; and 600–700 bars and 6–8 s—the post-processing injection pressure and time. The idea behind the manufacturing PA12 composites with 15 wt% of fibers is that such composites exist on the market in the form of filament and is used in additive manufacturing methods.

### 2.3. Characterization Methods

The microstructure of fibers and fabricated composites was assessed using a scanning electron microscope SEM TM3000 (Hitachi, Tokyo, Japan). The brittle fracture surface of composites made by thermoplastic and microsection of the thermoset matrix was investigated. Samples were fixed to carbon tape and coated with a gold/palladium mixture using POLARON SC7640 from Quorum Technologies Ltd., Lewes, UK (parameters: 100 s, 10 mA, 2 kV). Images were taken with an accelerating voltage of 5–15 kV.

The thermal decomposition of fibers and composites was examined in nitrogen and air employing the thermal analyzer TGA Q500 (TA Instruments Ltd., New Castle, DE, USA). The 10 mg samples were heated at 10 °C/min from room temperature to 1000 °C. The gas flow rate was 10 mL/min in the chamber and 90 mL/min in the furnace.

Differential scanning calorimetry (DSC) measurements were carried out using a DSC Q1000 (TA Instruments Ltd., USA). Samples of 5.0 ± 0.1 mg were placed in crucibles and then heated and cooled within the range of −80 °C to 240 °C for PA12-based composites and 180 °C for epoxy composites at a heating/cooling rate of 10 °C/min in a nitrogen atmosphere. The crystallinity amount (X_c_) of the PA12 composites was calculated from the following equation:

(1) $$X_{c}(\%)=\;\frac{∆H_{c}}{∆{H{}^{\circ}}_{m\;\;\;}(1-x)}\cdot 100\%$$

where Δ*H_c_* is the enthalpy of melting taken as the area under the melting peak from the second heating curve, Δ*H°_m_* is the melting enthalpy of 100% crystalline PA12, which is 209 J/g [20], and *x* is the weight fraction of added fibers.

The flexural properties of pressed samples were determined by a three-point bending test. Flexural strength and modulus were determined according to PN-EN ISO 178 standard [25] using a Shimadzu AGX-V equipped with a 100 kN load cell.

HDT tests according to ISO 75C [26] were carried out to determine the stiffness of the composites with recycled carbon fibers as the temperature increases. The specimens (size the same as for flexural tests) were analyzed in Zwick/Roell/Vicat Standard BVI-3300STD (Ulm, Germany).

Life cycle assessment was conducted to compare the environmental impacts of three end-of-life scenarios, namely landfill, mechanical, and chemical recycling. For this analysis, 1 kg of composite waste material, serving as the functional unit, was assessed. This waste material originated from the aviation industry, specifically from a military aircraft, and comprised an epoxy matrix containing both carbon fibers and glass fibers in a 90:10 ratio. LCA analysis was conducted using OpenLCA version 1.9, utilizing datasets from ecoinvent v3.6 system processes within open LCA, following the methodological framework described by [27]. The polymer matrix was represented by market for epoxy resin, liquid (GLO), which approximates diglycidyl ether of bisphenol-A (DGEBA) type resins commonly used in aerospace composite systems. Carbon fibers were mapped to carbon fiber, PAN-based, at plant (GLO), and glass fibers to glass fiber, at plant (GLO). Aluminum tooling was modeled using market for aluminum, primary, ingot (RoW). Regional energy inputs were described by market for electricity, medium voltage (PL) and heat, natural gas, at industrial furnace (PL), ensuring geographical consistency with production conditions. Where direct dataset analogs were unavailable, functionally equivalent proxies were adopted, consistent with best practice in life-cycle inventory modeling [28]. Release films and bagging materials were approximated by polyethylene and polyamide film markets, and generic solvents by market for acetone (GLO). The hardener mass was explicitly included as a separate flow, representing 0.20–0.33 kg per kg of epoxy resin, in accordance with stoichiometric ratios typical of amine-cured systems [29]. The base case employed market for ethanolamines (GLO) as a proxy for aliphatic amine hardeners, while market for diethylenetriamine (GLO) was used in sensitivity analysis to capture potential variation in precursor chemistry. Additives below 1% of total mass were excluded under a defined cut-off rule. Increasing total matrix mass by 2% changed the global warming potential by less than 3%, demonstrating low sensitivity to such uncertainty. Data quality was assessed using the ecoinvent pedigree matrix across reliability, completeness, and representativeness dimensions [27]. All major flows achieved a Data Quality Rating ≤ 3.5. Lower pedigree scores were assigned to solvent recovery stages due to limited foreground data availability. Uncertainty propagation through Monte Carlo simulation (*n* = 1000) generated 95% confidence intervals for dominant impact categories. Proxy validation using alternative resin and fiber datasets produced ≤ 5% variation in global warming potential, confirming that the selected proxies appropriately represent the studied materials within the model’s precision range.

The analysis employed system processes or life-cycle inventory, and the impact assessment was performed using the CML-IA baseline method. Data for mechanical and chemical recycling was provided from the consortium, while the landfilling and the rest of the stages were estimated and built up from the literature. To simplify the assessment, it was assumed that materials and production took place at the same site (i.e., material extraction stage, materials manufacture stage and product manufacture and production and assemblies take place on site) and that the only transportation inputs were for the transportation of the raw materials and landfill. The landfilling process was analyzed and assessed according to [22]. The system boundaries for the assessment were Cradle-to-Grave. The study applied a Cradle-to-Grave system boundary encompassing raw-material extraction, composite manufacturing, use, and end-of-life (EoL) management, in accordance with ISO 14040 [30] and 14044 guidelines [31]. Included processes comprised the production of fibers, resin, hardener, auxiliary consumables, and aluminum tooling; material transport; composite manufacturing and curing (LRTM/RTM); and operational transport equivalent to 300 km per 2.4 t of composite components within the Polish context. EoL scenarios incorporated landfilling, mechanical recycling, and chemical recycling (solvolysis), each accounting for internal energy use and residue handling. System-expansion credits were applied where recycled fibers demonstrably substituted virgin material of equivalent function, consistent with [28]. Excluded activities were limited to elements expected to contribute below materiality thresholds, consistent with ISO cut-off criteria [30]. Exclusions comprised capital goods (equipment, buildings, and tooling beyond the aluminium mold already included), administrative operations, packaging for intra-site transfers, and worker-related overheads. The combined mass and energy of all excluded processes remained below 5% of total life-cycle inputs. Biogenic carbon, land-use change, and product-use emissions were not applicable to the functional unit (1 kg composite waste). A sensitivity scenario including capital goods using machine tool, metalworking (GLO), and industrial hall (GLO) datasets altered global warming potential by less than 1%, confirming negligible influence. A complete inventory of inclusions, exclusions, and assumptions is available in the Supplementary Dataset (Supplementary Data S4) to ensure transparency and reproducibility.

## 3. Results and Discussion

### 3.1. Characterization of Composite Waste Fillers

**Figure 3:**
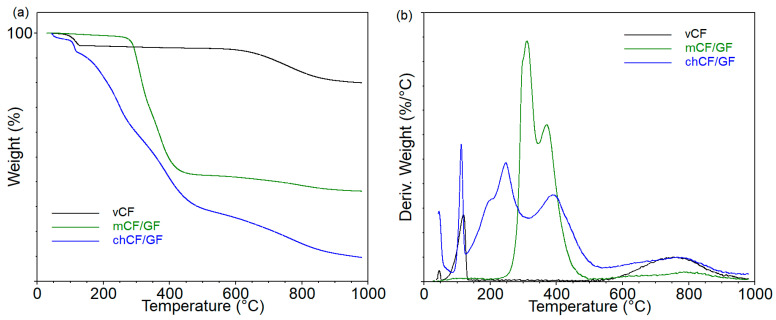
Mass loss (**a**) and derivative of mass loss (**b**) as functions of temperature from the thermogravimetric analysis of fillers.

The TGA results of the fillers (listed in Table 1) are shown in Figure 3, which displays the curves of mass loss and derivatives as a function of temperature, while the obtained data are summarized in Table 3. The analyses were carried out in an inert atmosphere and in the air to determine the residue yield in the presence of oxygen.

The temperature of 5% mass loss (T_5%_), corresponding to the beginning of the degradation process, was approximately 660 °C, 300 °C, and 130 °C for *v*CF, *m*CF/GF, and *ch*CF/GF, respectively. This means that used recycling methods, especially chemical recycling, significantly weaken the thermal stability of carbon fibers. The curves of derivatives of mass loss of *v*CF showed two distinct peaks (DTG1 and DTG5), which inform about the rate of material decomposition in the individual stages (Figure 3b). In the case of recycled fibers, the number of degradation stages increased to three for *m*CF/GF and to five for *ch*CF/GF. This is the effect of the presence of components, including an epoxy matrix from the composites subjected to the recycling process (DTG3), as well as substances used during the chemical recycling process (DTG2). In turn, DTG1 comes from releasing low-molecular-weight substances, probably absorbed by the fibers. The lowest rate of decomposition in individual stages was recorded for *v*CF, for which the lowest mass loss at the end of the analysis of 12% (in anaerobic conditions) was also observed. The yield of residues for *m*CF/GF and *ch*CF/GF for the analyses carried out in nitrogen was 62 and 46%, respectively. The opposite trend was observed in the case of analyses carried out in the air, for which the amount of residues for *v*CF fibers and after mechanical recycling was 2–3%, while after chemical recycling it was as much as 21%. This suggests that the procedures carried out during chemical recycling, or the presence of additional substances, stabilize the decomposition of fibers in aerobic conditions or promote the formation of new carbon structures.

### 3.2. Effect of the Composite Waste on the Epoxy System

**Figure 4:**
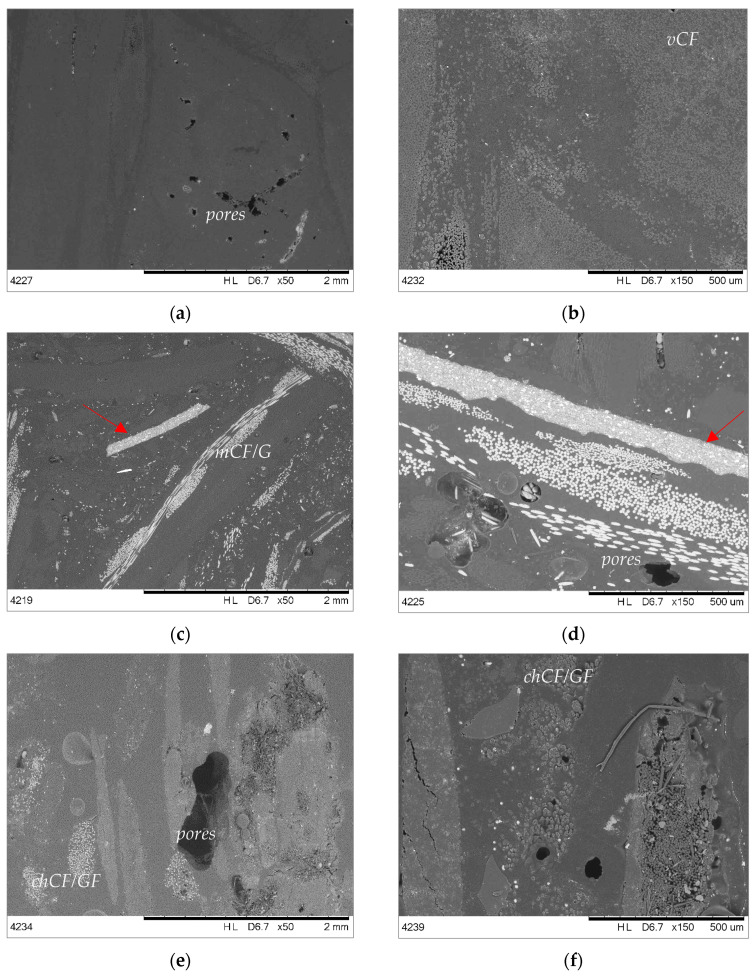
SEM images of epoxy composites’ microsection: EPOXY + 50% *v*CF (**a**,**b**); EPOXY + 50% *m*CF/GF (**c**,**d**); and EPOXY + 50 *ch*CF/GF (**e**,**f**).

The microsections of epoxy-based composites are presented in Figure 4. All epoxy-based composites have a few pores and discontinuities resulting from the manufacturing process, although EPOXY + 50% *v*CF/GF (Figure 4a,b) seems to have the most homogenous microstructure. The composite EPOXY + 50% *m*CF (Figure 4c,d) shows a layered arrangement of fibers with visible traces (white areas) of cured resin and paint (red arrows). The discontinuity of the structure with randomly arranged fibers is seen. The composite EPOXY + 50% *ch*CF/GF shows individual fibers and parts of resin coming from composite waste filler (see Table 1). In this case, the fibers were not milled before mixing with epoxy resin. Therefore, they are places where the mixture of CF/GF is concentrated. Nevertheless, the adhesion between epoxy and all types of fibers is satisfactory, even though the *m*CF/GF contains the traces of the composites (cured resin and paint), and *ch*CF/GF additionally lose the sizing from their surface after the solvolysis process.

**Figure 5:**
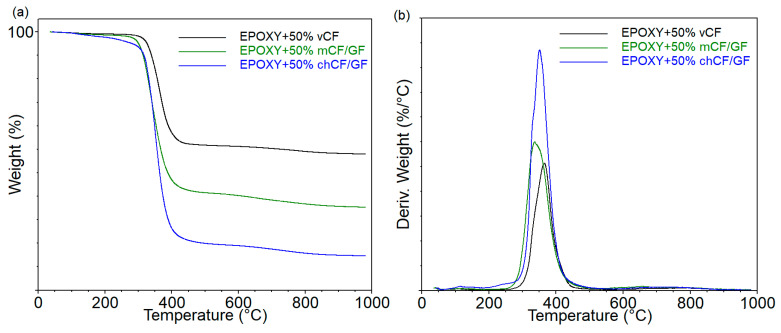
Mass loss (**a**) and derivative of mass loss as functions of temperature for epoxy-based composites (**b**).

Thermogravimetric analysis (TGA) was used to assess the thermal stability of the manufactured materials. The values obtained from the mass loss (TG) and mass loss derivatives (DTG) curves of the epoxy-based composites are presented in Table 4, while the graphs are presented in Figure 5.

The 5% weight loss temperature (T_5%_) of epoxy resin with virgin carbon fibers reached 328 °C, while adding *m*CF/GF or *ch*CF/GF decreased the T_5%_ to 303 °C and 290 °C, respectively. The decomposition process of EPOXY + 50% *v*CF has one main step, corresponding to the polymer’s degradation. The use of recycled fibers slightly altered the decomposition of composites. The DTG was shifted toward lower temperatures, and the degradation rate was higher than that of the EPOXY + 50% *v*CF. Moreover, the higher intensity of decomposition was accompanied by a decrease in the residue yield, so the lowest one was recorded for EPOXY + 50% *ch*CF/GF. The opposite trend was recorded for analysis conducted in the air.

**Figure 6:**
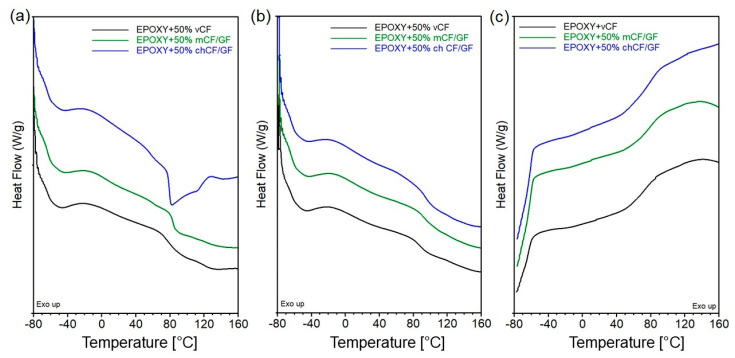
DSC thermograms representing: first heating (**a**); second heating (**b**); and cooling after first heating of epoxy-based composites (**c**).

To assess the correct course of the cross-linking process and describe the influence of interactions between the components, a differential scanning calorimetry (DSC) analysis was performed. The course of heat flow changes as a function of temperature in the first DSC heating cycle is presented in Figure 6.

The glass transition temperature, determined as an inflection from the first derivative of the heat flow curve, was 78, 84 and 79 °C for epoxy with *v*CF, *m*CF/GF and *ch*CF/GF, respectively. Using recycled fibers resulted in a slight increase in the glass transition temperature values, especially in the case of *m*CF/GF. The increased glass transition temperature can be attributed to the hindering of the macromolecular chains’ motions by high-surface area fibers and/or to hydrogen bonding between hydroxyl groups of epoxy and groups present on the filler’s surface, limiting the macromolecular motions at elevated temperatures [23]. Moreover, no additional exothermic processes above the glass transition were observed, demonstrating that the resin was completely cured [32].

Results of selected mechanical parameters of the three types of composites presented in Table 5 show the influence of the recycling method of cured composites on flexural strength, flexural modulus, and heat deflection temperature (HDT). The idea was to use the composite waste directly in the hot-pressing process and to analyze the effect on the epoxy resin. Therefore, used fillers (*v*CF, *m*CF/GF and *ch*CF/GF) have different sizes, as marked in Table 2.

As expected, the composites containing fibers after recycling possess lower flexural parameters than the reference material containing *v*CF. As a result, EPOXY + 50% *m*CF/GF fibers exhibit relatively low mechanical parameters, closer to composites reinforced with particle-like fillers, resulting in composites with isotropic mechanical behavior. The flexural modulus and strength were lowered by about 46% and 25% in comparison to epoxy reinforced with *v*CF. At the same time, the composite reinforced with *ch*CF/GF exhibits almost the same mechanical properties as composites containing virgin fibers. Here the flexural modulus and strength were decreased by about 12% and 15% in relation to EPOXY with *v*CF, which is a relatively small drop. This finding implies that *ch*CF/GF with chemically removed matrix residues and sizing exhibit better chemical affinity to epoxy resin than *m*CF/GF containing epoxy residues on their surface. The fracture behavior of selected specimens also supports the above observations (see Figure S3 in the Supplementary Materials). Composites with *m*CF/GF composite do not break at one point, showing no step-fracture that is typically observed in the case of fiber-reinforced materials. Both samples, *ch*CF/GF and *v*CF, follow an anisotropic fracture pattern. From thermal point of view, HDT values for investigated composites were almost the same for EPOXY containing *v*CF and *m*CF/GF, while for EPOXY + 50% *ch*CF/GF the HDT is significantly decreased. On the one hand it can be caused by the lack of sizing on the fiber after chemical recycling and thus by decreased interfacial bonding between the polymer and the filler.

Hwand et al. [33] found that dual-sized carbon fibers significantly contributed to improving the heat deflection temperature of ABS compared with carbon fibers having a single sizing on their surface. On the other hand, higher mechanical properties of EPOXY with *ch*CF/GF can be associated with the form of the fillers, which occurs as small pieces of mats/fabrics (Table 5) that are more resistant to breakage.

### 3.3. Effect of the Composite Waste on the Thermoplastic Matrix

**Figure 7:**
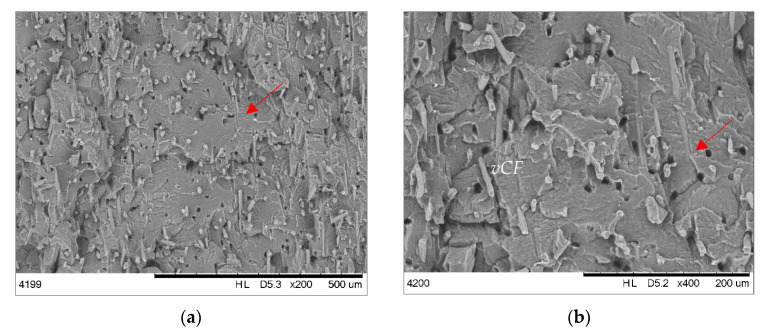

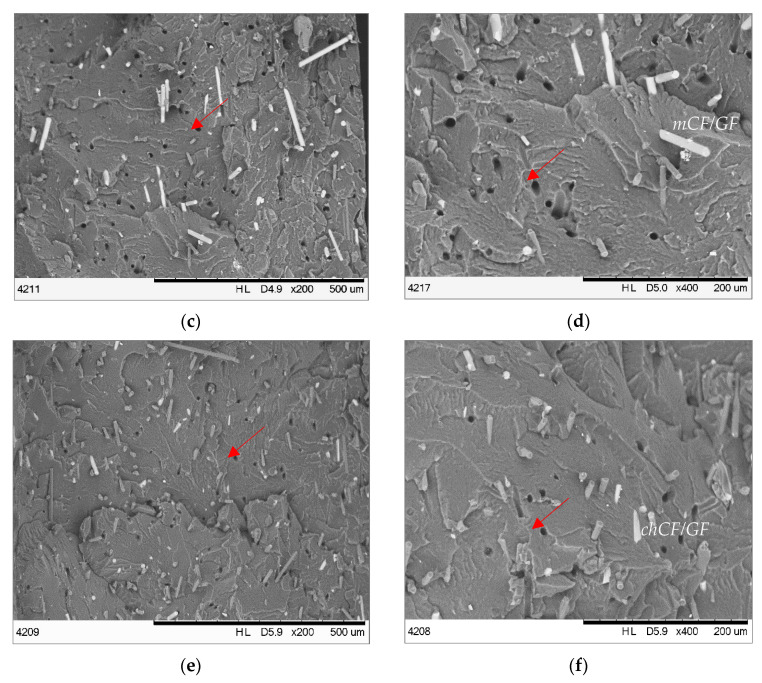
SEM images of thermoplastic composites’ microsection: PA12 + 15% *v*CF (**a**,**b**); PA12 + 15% *m*CF/GF (**c**,**d**); and PA12 + 15% *ch*CF/GF (**e**,**f**).

SEM images of the brittle surface of PA12-based composite are presented in Figure 7. It can be seen that all composites possess a similar microstructure where single fibers are distributed homogeneously in the PA12 matrix. However, there are visible holes (red arrows) related to pulling out the fibers from the polymer matrix formed during the preparation of the brittle fracture surface. It reflects poor interfacial adhesion between all types of fillers and matrix [9]. In the case of PA12, the adhesion strength is strongly related to the type of carbon fibers. Rosso et al. [34] proved that oxidized carbon fibers show higher affinity to PA12 than fibers with chemical sizing. Hence, the surface chemistry of the fibers coming from various recycling approaches plays an important role in the reinforcement mechanism. It should be noted that in the case of composites containing *m*CF/GF and *ch*CF/GF the presence of epoxy and paint traces does not negatively affect the overall composite quality. All components are well mixed, which is related to the chemical compatibility between epoxy and polyamides.

**Figure 8:**
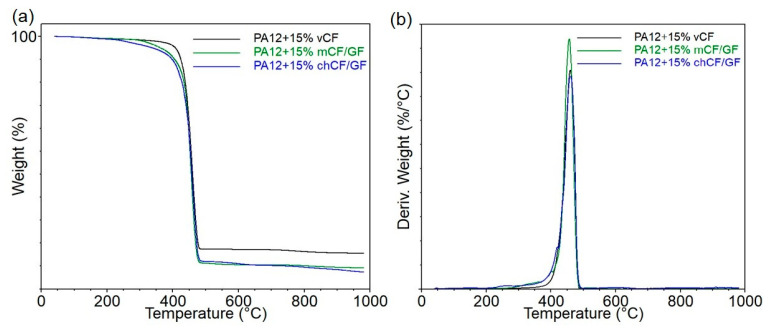
Mass loss (**a**) and derivative of mass loss as functions of temperature from the thermogravimetric analysis of PA12-based composites (**b**).

Thermogravimetric analysis (TGA) was used to assess the thermal stability of the manufactured materials. The values obtained from the mass loss (TG) and mass loss derivatives (DTG) curves of the PA12-based composites are presented in Table 6, while the graphs are presented in Figure 8.

The onset temperature (T_5%_) of PA12 with *v*CF was 411 °C and decreased for *m*CF/GF and *ch*CF/GF by 41 °C and 62 °C, respectively. Although a slight peak in the case of composites with recycled fibers was observed on DTG curves around 400 °C, the most intense degradation of PA appeared at approx. 460 °C. No significant changes or linear dependence in the DTG occurrence and degradation rate occurred, which can be attributed to the small size of the analyzed samples. However, due to the recycling processes applied, the residual mass decreased from 14% (PA12 + 15% *v*CF) to almost 7% (PA12 + 15% *ch*CF/GF) for analysis performed in nitrogen. Similarly to the epoxy composites, the highest residue yield in the air was obtained for fibers after mechanical recycling.

**Figure 9:**
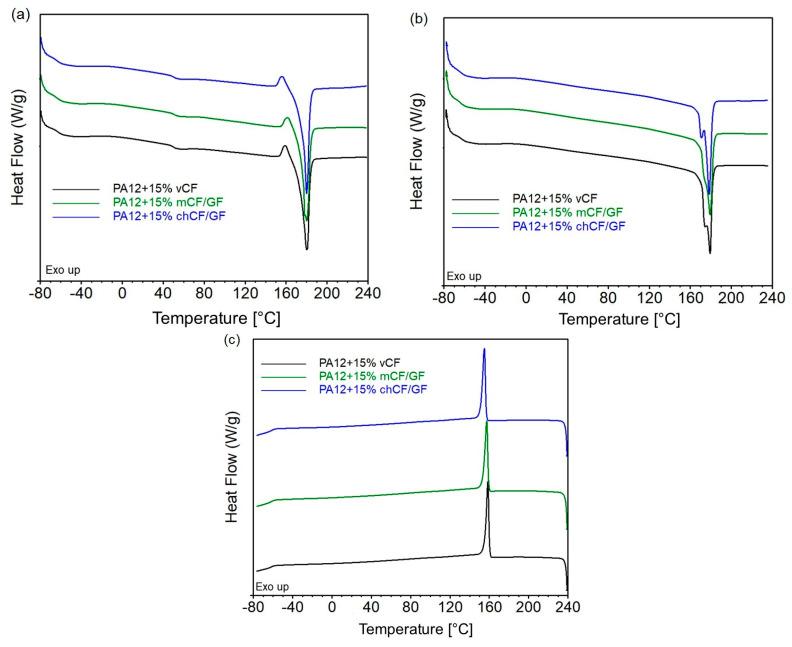
DSC thermograms representing first heating (**a**), second heating (**b**), and cooling after first heating (**c**) of PA12-based composites.

The DSC thermograms for PA12-based composites are presented in Figure 9, while the determined characteristic parameters are collected in Table 7. Polyamide 12 is characterized by various crystalline polymorphs and can crystallize in four different crystallographic forms [35]. Comparing the results of the first and second heating, a different course of the DSC curves can be noted. The first heating is significant for correlating the structure of PA12 and its composites with their functional properties, containing information about processing influence on polymer structure. It can be assumed that intensive cooling of the materials during shaping resulted in a heterogeneous crystal structure. In the first heating, all materials have a glass transition temperature (T_g_) of approximately 50 °C and an exothermic peak associated with the reorganization of less thermodynamically stable imperfect crystals. The observed double endothermal peak on the second heating curve is related to the presence of both α- and γ-form crystallite for PA12 containing *v*CF and *m*CF/GF, as it was described for metal oxide-filled PA12 composites [36]. This effect is less visible for PA12 + 15% *ch*CF/GF and is only noted as an inflection of the narrower endothermic peak. The addition of recycled fillers caused a slight change in crystallization temperature (T_c_) compared to the composite series reinforced with *v*CF. It cannot be ruled out that the chemical recycling of the fibers resulted in an increased number of hydroxyl groups on the surface of the fillers as a result of the dissolution of the polymer and the removal of the previously excessive sizing, which resulted in the possibility of additional interactions between OH on the surface of the glass and carbon fibers with the amide group of polyamide [37,38]. It leads to the conclusion that under technological process conditions, it is possible to obtain a similar structure of materials regardless of the origin of the fibers (virgin/recycled), despite the apparent differences between the thermal behavior of materials crystallized under controlled conditions.

The influence of virgin and recycled fibers on the mechanical properties of PA12 was analyzed in a three-point bending test. Based on the results collected in Table 8, the composites with *v*CF resulted in the highest flexural strength and modulus. In relation to PA12 + 15% *v*CF, the composites containing *m*CF/GF and *ch*CF/GF have lowered flexural properties of about 36% and 29%, respectively. In general, higher brittleness obtained for PA12 with recycled fibers can be related to changing the surface properties after mechanical and chemical recycling. Various authors proved that recycled fibers have modified morphology (roughness), types of surface functional properties, as well as a potential layer of char residue obtained after the pyrolysis process which all affect the wettability of the fiber by polymer resin [39]. On the one hand, Hendlmeier et al. [40] described that composites of PA6 with epoxy and polyamide sizing short carbon fibers strongly affect the flexural modulus and strength. On the other hand, it was described that in some cases recycled fibers with removed sizing after pyrolysis (thermal recycling) or solvolysis process (chemical recycling) resulted in higher mechanical properties after mixing with thermoplastic polymer. Burn et al. [41] explained that recycled carbon fibers have a rougher surface that helps to create a mechanical keying with the polymer. Furthermore, the presence of cured epoxy and paint traces added together with *m*CF/GF negatively affects the adhesion between PA12 matrix and fibers resulting in the lowest mechanical properties among all studied materials.

### 3.4. Life Cycle Assessment

**Figure 10:**
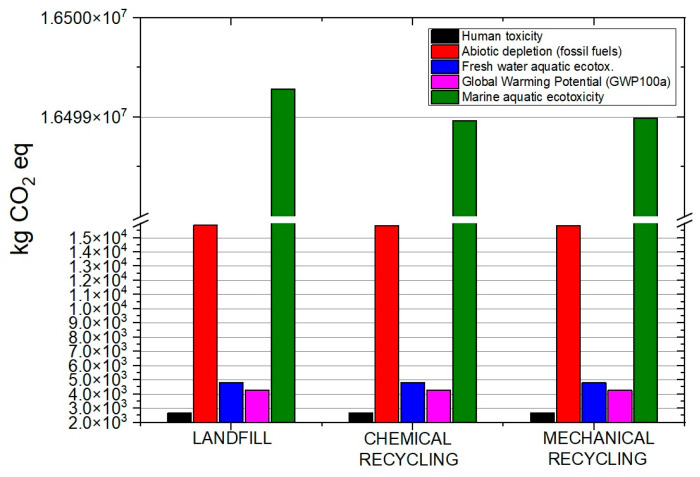
Environmental impact of the three processes.

**Figure 11:**
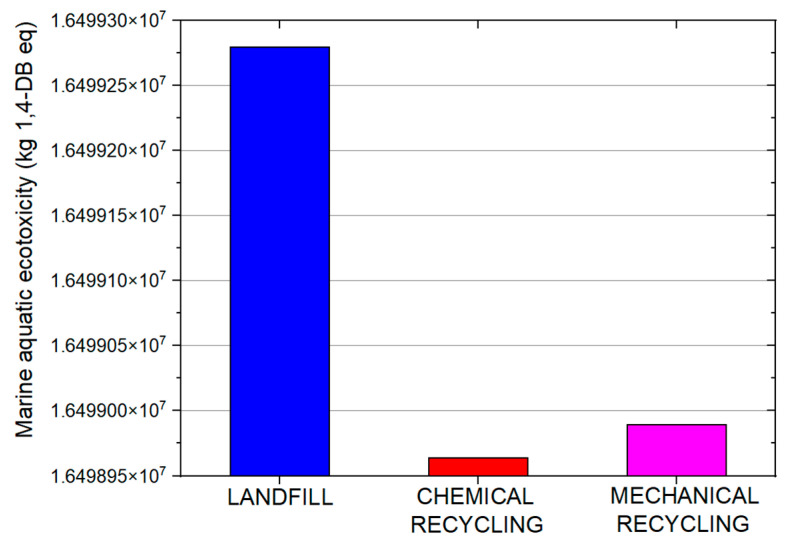
Marine aquatic ecotoxicity environmental impact of the three analyzed processes.

**Figure 12:**
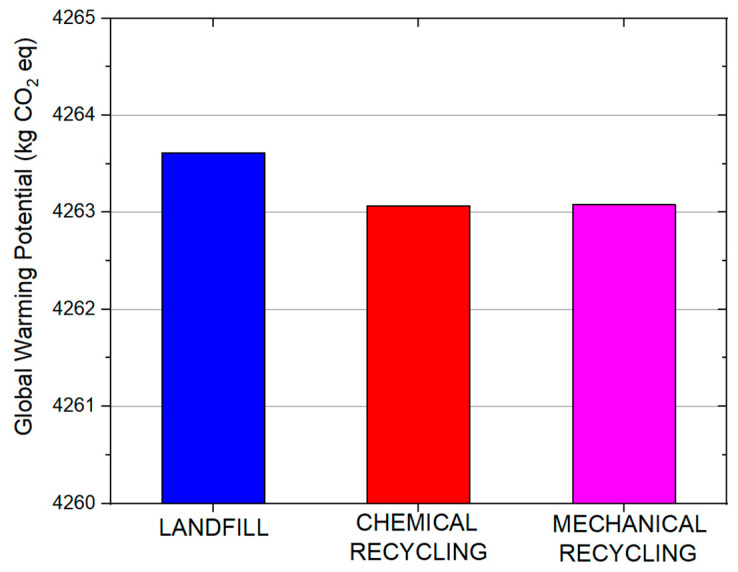
Global warming environmental impact of the three analyzed processes.

The life cycle assessment analysis indicated that all three processes affected the environmental indicators under consideration (Figure 10). Several life-cycle impact indicators were calculated including, human toxicity, abiotic depletion, marine aquatic ecotoxicity, global warming, and freshwater aquatic ecotoxicity. The two primary impact indicators were marine aquatic ecotoxicity (Figure 11) and global warming potential (Figure 12). All processes significantly impacted both indicators, with the landfill scenario appearing to have the greatest impact. Another influential indicator was abiotic depletion (fossil fuels), which is related to depletion of non-renewable resources or abiotic materials in the natural environment. In this case, all three processes had a large impact on the abiotic depletion. Finally, the last indicator with substantial impact was the freshwater aquatic ecotoxicity which refers to the adverse effects of toxic substances on freshwater ecosystems and the organisms living in them.

The comparison of the impact of landfilling, chemical recycling, and mechanical recycling processes on the two key environmental indicators is depicted separately in order to evaluate the differences in the values. As can be seen, landfilling exhibited a significantly higher impact when compared with the two recycling processes. The lowest impact in terms of marine aquatic ecotoxicity and global warming was exhibited by the chemical recycling process.

Considering the three scenarios under LCA evaluation, the similarity in environmental performance between the mechanical and chemical recycling scenarios was indicated by the proximity of their values. Although landfilling is the most labor-efficient and cost- effective end-of-life management option, it demonstrated the highest environmental impact across assessed categories. Results indicated a proximity of the environmental indicators for the mechanical and chemical recycling scenarios; environmental impacts indicated comparable influence on assessed categories. In the chemical recycling scenario, evaluating chemical impacts and implementing cleaner production techniques is key for reducing environmental footprints. Strategies like material substitution and energy-efficient practices can reduce burdens in mechanical recycling. The landfilling scenario indicates that, while cost-effective, it contributes significantly to marine and freshwater aquatic ecotoxicity, emphasizing the need for waste management improvements. To approach a more effective waste management strategy, collaboration among stakeholders and the adoption of circular economy principles is essential. As a whole, strengthening monitoring, focusing on regulatory measures, incentivizing sustainable practices, and integrating life cycle assessments early on can enhance environmental performance in all of the processes.

## 4. Conclusions

This study investigated the mechanical and chemical recycling of complex aerospace composite waste containing mixed carbon and glass fibers to evaluate their potential for reuse in thermosetting and thermoplastic polymer matrices. Chemical solvolysis using nitric acid successfully reclaimed these fibers, although the process significantly reduced the 5% mass loss temperature of the fibers from 662 °C for virgin material to 131 °C. In contrast, mechanical recycling resulted in a fiber thermal stability of 297 °C. When the rCF/rGF mixture was integrated into an epoxy matrix at a 50 wt% loading, the chemically recycled fibers achieved a flexural modulus of 9.9 GPa and a strength of 112 MPa, representing a relatively small decline from the 11.3 GPa and 132 MPa recorded for virgin carbon fibers. Mechanically recycled rCF/rGF in the same epoxy system showed a much larger drop, reaching only 6.1 GPa in modulus and 98.0 MPa in strength. Heat deflection temperatures for these epoxy composites were 83 °C for virgin carbon fibers and 82 °C for rCF/rGF after mechanical recycling but dropped to 71 °C for the chemically recycled version. In thermoplastic polyamide 12 modified with 15 wt% of rCF/rGF, chemically recycled fibers yielded a flexural modulus of 2.14 GPa and a strength of 67.7 MPa, whereas addition of fibers after mechanical recycling resulted in lower values of 1.92 GPa and 63.9 MPa, respectively. The thermal stability for these polyamide composites was 411 °C for virgin fibers, 370 °C for fibers after mechanical recycling, and 349 °C for fibers after chemical recycling. Crystallinity measurements for the polyamide composites showed that addition of fibers after chemical recycling resulted in the highest value at 27.5%, compared to 26.0% for virgin fibers and 25.4% for fibers after mechanical recycling. Life cycle assessment based on a functional unit of 1 kg of composite waste confirmed that both recycling pathways are significantly more sustainable than landfilling, specifically with regard to global warming potential and marine aquatic ecotoxicity. These numerical results prove that while recycled rCF/rGF fibers exhibit lower properties than virgin materials, they maintain sufficient performance for less demanding structural applications, such as for interior parts in automotive or rail interiors, covers, construction formwork, protective panels, or recreational products.

## Figures and Tables

**Table 1:** Macro- and microstructure, size, and designation of the fibrous fillers used.

Fibers Type	Macrostructure	Microstructure
Virgin carbon fibers (*v*CF) with length of 13 mm for hot-pressing	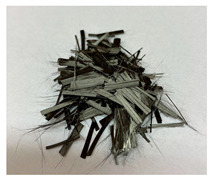	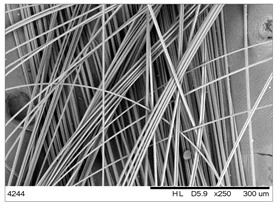
Virgin carbon fibers *(v*CF) with length of 0.25 mm for extrusion	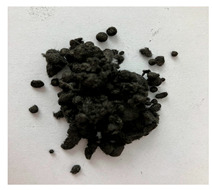	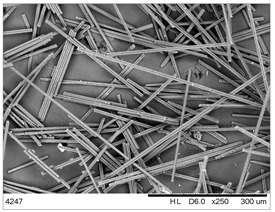
After mechanical recycling (*m*CF/GF) for hot-pressing	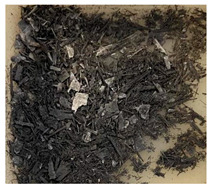	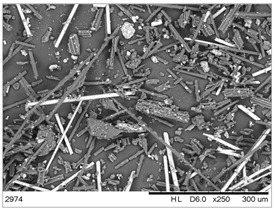
After mechanical recycling (*m*CF/GF) and milling 0.25 mm for extrusion	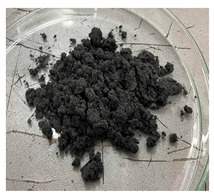	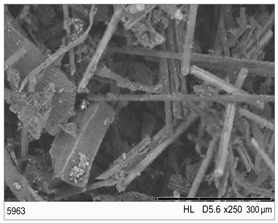
After chemical recycling (*ch*CF/GF) for hot-pressing	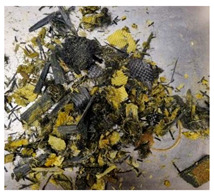	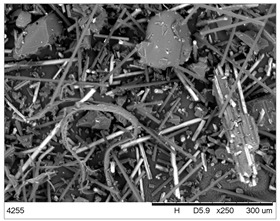
After chemical recycling (*ch*CF/GF) and milling 0.25 mm for extrusion	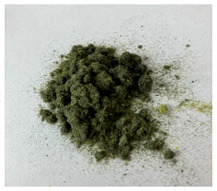	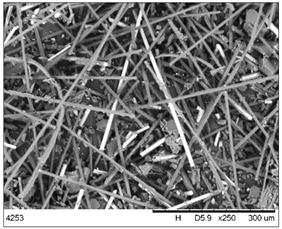

**Table 2:** The list of manufactured thermoset and thermoplastic composites.

Samples Designation	Type of Filler (Listed in Table 1)	Filler Content [wt%]
EPOXY + 50% *v*CF	Virgin CF 13 mm	50
EPOXY + 50% *m*CF/GF	A mixture of CF/GF after mechanical recycling	
EPOXY + 50% *ch*CF/GF	A mixture of CF/GF after chemical recycling	
PA12 + 15% *v*CF	Virgin CF 0.25 mm	15
PA12 + 15% *m*CF/GF	A mixture of CF/GF after mechanical recycling	
PA12 + 15% *ch*CF/GF	Mixture of CF/GF after chemical recycling	

**Table 3:** The results from thermogravimetric analysis for used fibers.

FIBER TYPE	T_5%_ °C	DTG1 °C %/min	DTG2 °C %/min	DTG3 °C %/min	DTG4 °C %/min	DTG5 °C %/min	Residue at 1000 °C in Inert Atmosphere	Residue at 1000 °C in Air
*v*CF	662	118 0.09	-	-	-	755 0.04	88.0	3.2
*m*CF/GF	297	-	-	310 0.36	371 0.23	785 0.01	61.8	2.2
*ch*CF/GF	131	113 0.20	204 0.12	247 0.18	390 0.13	768 0.04	45.8	20.8

**Table 4:** The results from TGA of epoxy-based composites.

Composite	T_5%_ °C	DTG1 °C; %/min	Residue at 975 °C in Inert Atmosphere	Residue at 975 °C in Air
EPOXY + 50% *v*CF	328	365; 0.65	52.7	1.4
EPOXY + 50% *m*CF/GF	303	337; 0.76	32.1	7.8
EPOXY + 50% *ch*CF/GF	290	351; 1.24	13.3	2.1

**Table 5:** Flexural modulus, flexural strength, and heat deflection temperature (HDT) of epoxy-based composites.

Composite	Flexural Modulus [GPa]	Flexural Strength [MPa]	HDT [°C]
EPOXY + 50% *v*CF	11.3 ± 0.8	132 ± 0.9	83 ± 0.6
EPOXY + 50% *m*CF/GF	6.1 ± 0.3	98.0 ± 1.1	82 ± 0.1
EPOXY + 50% *ch*CF/GF	9.9 ± 0.4	112 ± 1.2	71 ± 0.3

**Table 6:** The results of thermogravimetric analysis of PA12-based composites.

Composite	T_5%_ °C	DTG1 °C; %/min	DTG2 °C; %/min	Residue at 1000 °C in Inert Atmosphere	Residue at 1000 °C in Air
PA12 + 15% *v*CF	411	-	459; 2.06	14.1	0.6
PA12 + 15% *m*CF/GF	370	402; 0.17	467; 2.36	8.4	8.3
PA12 + 15% *ch*CF/GF	349	419; 0.37	460; 2.00	6.7	5.0

**Table 7:** DSC thermal parameters of PA12-based composites.

Composite	T_g1_ [°C]	T_m1_ [°C]	T_c_ [°C]	T_m2_ [°C]	X_c1_ [%]	X_c2_ [%]
PA12 + 15% *v*CF	49	180	159	174/179	26.0	24.6
PA12 + 15% *m*CF/GF	51	178/180	157	179	25.4	23.8
PA12 + 15% *ch*CF/GF	49	180	155	170/178	27.5	23.7

**Table 8:** Flexural modulus and flexural strength of PA12-based composites.

Composite	Flexural Modulus [GPa]	Flexural Strength [MPa]
PA12 + 15% *v*CF	3.03 ± 0.20	85.1 ± 0.84
PA12 + 15% *m*CF/GF	1.92 ± 0.08	63.9 ± 0.90
PA12 + 15% *ch*CF/GF	2.14 ± 0.07	67.7 ± 0.61

## Data Availability

The raw data supporting the conclusions of this article will be made available by the authors on request.

## Supplementary Materials

The following supporting information can be downloaded at https://www.mdpi.com/article/10.3390/ma19030534/s1, Figure S1. Chemical recycling of the CGFRP specimens: laboratory set-up for solvolysis process (a); recycled fibers (b); obtained extract (c). Figure S2. Epoxy-based composites specimens produced by hot-pressing: epoxy + 50 wt% *v*CF with 13 mm diameter (a); epoxy + 50 wt% *m*CF/GF (b); and epoxy + 50 wt% c*h*CF/GF (c). PA12-based composite specimens: PA12 + 15 wt% *v*CF with 0.25 mm diameter (d); PA12 + 50 wt% *m*CF/GF (e); and PA12 + c*h*CF/GF with 0.25 mm (f). Figure S3. Flexural stress–strain curves for the EPOXY-based composites containing *v*CF, *m*CF/GF, and *ch*CF/GF. LCA Assumption S4. References [13,14,16,18,19,22,27,28,30,31,42] are cited in the Supplementary Materials.
