# Supplementary material for: Recycling and Reusing of Waste Aircraft Composites in Thermoplastic and Thermoset Matrices
Source: Materials (Basel). 2026 Jan 29;19(3):534. doi: 10.3390/ma19030534 (PMC12898532; doi:10.3390/ma19030534)
Supplement: Supplementary file 1 [file materials-19-00534-s001.zip › materials-4097042-supplementary.pdf]

Supplementary Materials

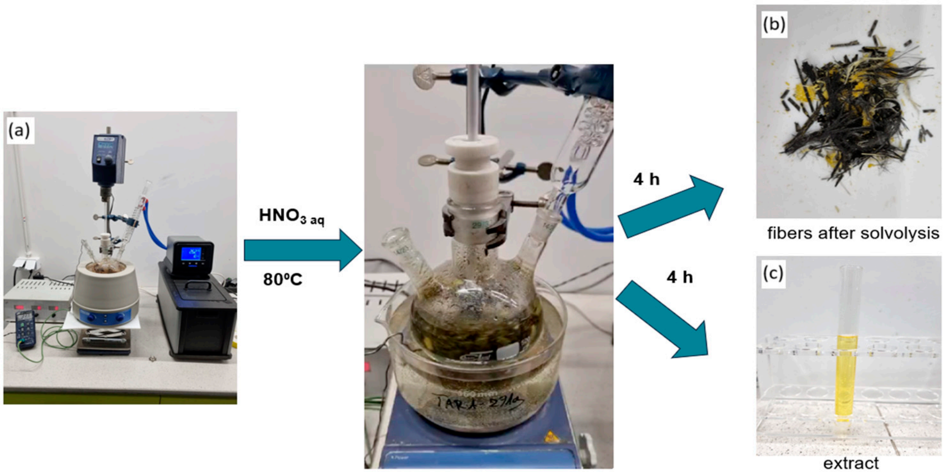

Figure S1. Chemical recycling of the CGFRP specimens: (a) laboratory set-up for solvolysis process, (b) recycled fibers, (c) obtained extract.

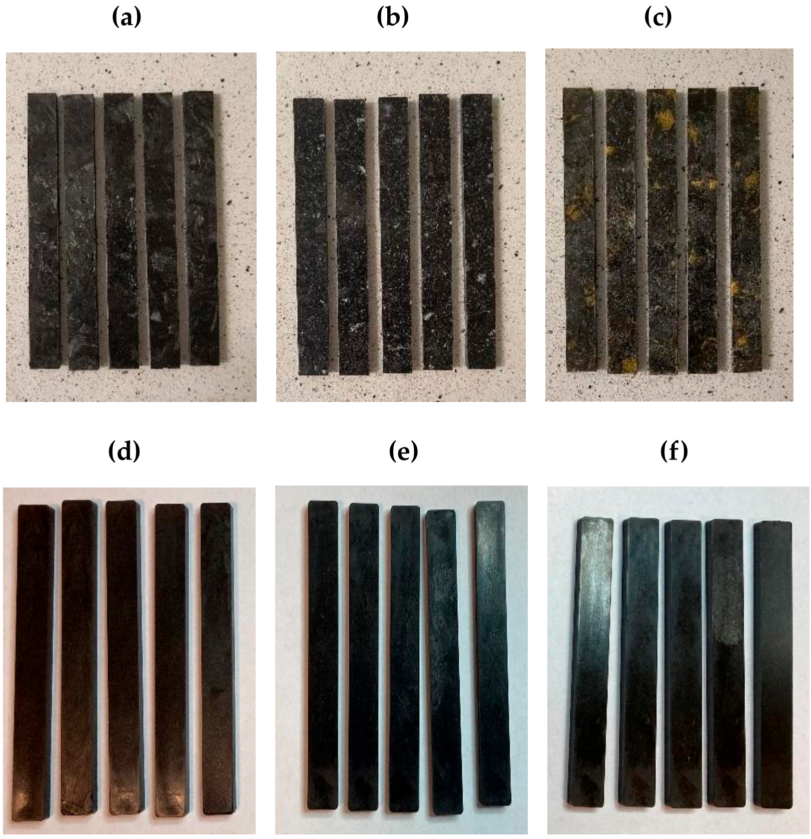

Figure S2. Epoxy-based composites specimens produced by hot-pressing: epoxy+50wt% *v*CF with 13 mm diameter (a); epoxy+50wt% *m*CF/GF (b) and epoxy+50 wt% *ch*CF/GF (c). PA12-based composite specimens: PA12+15wt% *v*CF with 0.25 mm diameter (d); PA12+50wt% *m*CF/GF (e) and PA12+*ch*CF/GF with 0.25 mm (f).

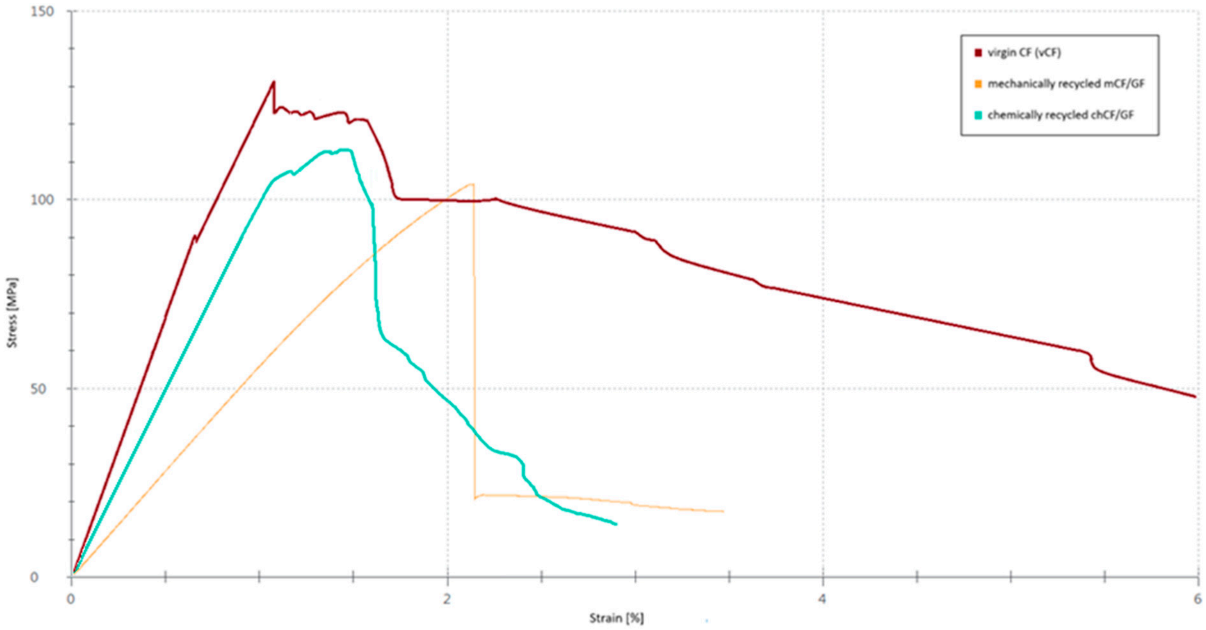

Figure S3. Flexural stress-strain curves for the EPOXY-based composites containing. *v*CF, *m*CF/GF, *ch*CF/GF.

Life cycle inventory (LCI) assumptions and modelling choices S4.

In the present study, the assumption that material production and composite manufacturing occur on the same site was introduced as a modeling simplification to reduce system complexity and avoid introducing poorly constrained transport inventories. This is in line with ISO 14044 recommendations for comparative LCAs when transport is not expected to be a dominant contributor. This assumption does not imply zero transportation but rather reflects a foreground system where internal logistics are negligible compared to material and energy flows.

Transportation of raw materials and waste to landfill was explicitly included (each in the appropriate stage) using regionally consistent datasets. Regional energy mixes were fully accounted for using Polish electricity and heat datasets, reflecting actual production conditions. Solvent use in chemical recycling was modelled explicitly, and solvent recovery was conservatively treated with partial recovery assumptions based on literature ranges.

To address the reviewer's concern, we have:

- Clarified the scope and intent of the "same site" assumption in the main text, and
- Added an explicit list of LCA assumptions

We strongly believe that these additions improve transparency as they are not altering the comparative conclusions of the study.

Life cycle assessment (LCA) was conducted in accordance with ISO 14040 and ISO 14044 standards to compare landfilling, mechanical recycling, and chemical recycling of aircraft composite waste. The functional unit was defined as 1 kg of composite waste, composed of an epoxy matrix reinforced with carbon and glass fibers (90:10 mass ratio), representative of post-production aerospace composite scrap.

A foreground system simplification was applied in which material production, composite manufacturing, and recycling operations were modelled as co-located. This assumption does not imply the absence of transportation but reflects a common modelling choice in comparative LCAs of recycling technologies, intended to avoid introducing speculative logistics data when transport is not expected to dominate environmental impacts [1–3]. The assumption was applied consistently across all scenarios to preserve comparability.

Transportation was explicitly included where well defined, namely for raw material supply and waste transport to landfill, using standard European freight transport datasets. Sensitivity checks confirmed that reasonable variations in transport distances did not affect the relative ranking of the end-of-life scenarios, consistent with findings reported for CFRP and GFRP recycling systems [2,4].
Electricity and thermal energy inputs were modelled using region-specific Polish datasets (medium-voltage electricity mix and industrial natural gas heat), ensuring geographical consistency with the experimental and industrial context. Regionalized energy modelling is recognized as a critical parameter in LCAs of energy-intensive composite processing [5,6].
Chemical recycling was modelled with explicit inclusion of solvent and reagent consumption. Nitric acid demand was based on experimental conditions. Solvent recovery was conservatively modelled assuming partial recovery, reflecting laboratory-scale and early pilot-scale solvolysis processes reported in the literature [7–9]. Residual solvent losses were allocated to waste treatment. Mechanical recycling included electricity demand and material losses derived from foreground data.
Where exact datasets were unavailable, functionally equivalent proxies from ecoinvent v3.6 were used following established LCA practice [5]. Cut-off criteria excluded flows contributing less than 1% of total mass or energy, with cumulative exclusions remaining below 5%. Capital goods and administrative processes were excluded, as their contribution to total impacts was shown to be negligible in sensitivity analysis.
The principal modelling assumptions employed in this study are summarized in Table 1. These assumptions follow ISO guidance for comparative assessment and are consistent with composite recycling technologies LCA literature.

Table 1. Key modelling assumptions and justification

Aspect	Assumption	Note
Production location	Material production, manufacturing, and recycling treated as co-located	This is quite common (for simplification purposes) in comparative LCAs when transport is non-dominant [1–3]
Transportation	Included for raw materials and landfill only	Transport shown to be secondary contributor in CFRP LCAs [2,4]
Electricity supply	Polish grid mix (medium voltage)	Regional energy mix strongly influences GWP indicator [5,6]
Thermal energy	Industrial natural gas heat (PL)	Representative for composite curing and recycling [6]
Solvent recovery	Partial recovery assumed	Conservative assumption aligned with solvolysis literature [7–9]
Recycling credits	System expansion for substituted virgin fibers	Recommended for recycling studies [2,10]
Cut-off criteria	<1% mass or energy per flow	ISO-compliant, cumulative <5% [11]
Capital goods	Excluded	Demonstrated negligible influence in sensitivity checks

It is important to clarify here that uncertainty associated with transport distances, energy supply, and solvent recovery was addressed through conservative assumptions and sensitivity analysis. Transport distances were varied within realistic European ranges, confirming that transport remained a secondary contributor relative to material and energy inputs, consistent with previous CFRP and GFRP recycling studies [2,4]. Regional energy supply was identified as a key driver of absolute impacts, particularly for global warming potential. However, since identical energy datasets were applied across all scenarios, comparative conclusions remained robust. This behavior aligns with observations reported in LCAs of composite manufacturing and recycling [5,6]. Solvent recovery efficiency constituted the largest source of foreground uncertainty in the chemical recycling scenario. Conservative recovery rates were therefore applied, reflecting reported laboratory and pilot-scale performance for nitric-acid-based solvolysis [7–9].

Overall, the applied assumptions comply with ISO 14040/44 requirements for comparative LCA and are consistent with methodological choices reported in the literature on composite recycling and end-of-life assessment [2,3,10].

References to S4

1. ISO 14044:2006. *Environmental management — Life cycle assessment — Requirements and guidelines*.
2. Witik, R.A.; et al. Carbon fibre reinforced composite waste: An environmental assessment of recycling, energy recovery and landfilling. *Compos. Part A* 2013, 49, 89–99.
3. Pimenta, S.; Pinho, S.T. Recycling carbon fibre reinforced polymers for structural applications. *Waste Management* 2011, 31, 378–392.
4. La Rosa, A.D.; Banatao, D.R.; Pastine, S.J.; Latteri, A.; Cicala, G. Recycling Treatment of Carbon Fibre/Epoxy Composites: Materials Recovery and Characterization and Environmental Impacts through Life Cycle Assessment. *Compos. Part B Eng.* **2016**, *104*, 17–25.
5. Wernet, G.; Bauer, C.; Steubing, B.; Reinhard, J.; Moreno-Ruiz, E.; Weidema, B.P. The Ecoinvent Database Version 3 (Part I): Overview and Methodology. *Int. J. Life Cycle Assess.* **2016**, *21*, 1218–1230.
6. Kočí, V.; Picková, E. Life cycle perspective of epoxy resin use. *Pol. J. Environ. Stud.* 2019, 29, 653–667.
7. Zhao, Q.; Jiang, J.; Li, C.; Li, Y. Efficient Recycling of Carbon Fibers from Amine-Cured CFRP Composites under Facile Condition. *Polym. Degrad. Stab.* **2020**, *179*, 109268.
8. Sakai, A.; Kurniawan, W.; Kubouchi, M. Chemical Recycling of CFRP in an Environmentally Friendly Approach. *Polymers* **2024**, *16*, 143.
9. Shetty, S.; et al. Recycling CFRP in subcritical acetic acid. *Heliyon* 2022, 8, e12242.
10. European Commission – JRC. ILCD Handbook: Recommendations for Life Cycle Impact Assessment.
11. ISO 14040:2006. *Environmental management — Life cycle assessment — Principles and framework.*
